# Scalable Preparation of Cellulose Nanofibers from Office Waste Paper by an Environment-Friendly Method

**DOI:** 10.3390/polym13183119

**Published:** 2021-09-15

**Authors:** Deyuan Huang, Haoqun Hong, Weilong Huang, Haiyan Zhang, Xiaobin Hong

**Affiliations:** 1 School of Materials and Energy, Guangdong University of Technology, Guangzhou 510006, China; 2111902033@mail2.gdut.edu.cn (D.H.); pshqhong@gmail.com (H.H.); 3119003499@mail2.gdut.edu.cn (W.H.); hyzhang@gdut.edu.cn (H.Z.); 2 School of Mechanical and Automotive Engineering, South China University of Technology, Guangzhou 510640, China

**Keywords:** waste paper, TEMPO, cellulose nanofiber (CNF), recycling, bleaching

## Abstract

Waste paper is often underutilized as a low-value recyclable resource and can be a potential source of cellulose nanofibers (CNFs) due to its rich cellulose content. Three different processes, low acid treatment, alkali treatment and bleaching treatment, were used to pretreat the waste paper in order to investigate the effect of different pretreatments on the prepared CNFs, and CNFs obtained from bleached pulp boards were used as control. All sample fibers were successfully prepared into CNFs by 2,2,6,6-tetramethyl-piperidine-1-oxyl (TEMPO) oxidation. It was quite obvious that the bleached CNFs samples showed dense fibrous structures on a scanning electron microscopy (SEM), while needle-like fibers with width less than 20 nm were observed on a transmission electron microscopy (TEM). Meanwhile, the bleaching treatment resulted in a 13.5% increase in crystallinity and a higher TEMPO yield (e.g., BCNF, 60.88%), but a decrease in thermal stability. All pretreated CNFs samples showed narrow particle size distribution, good dispersion stability (zeta potential less than −29.58 mV), good light transmission (higher than 86.5%) and low haze parameters (lower than 3.92%). This provides a good process option and pathway for scalable production of CNFs from waste papers.

## 1. Introduction

With the increasing calls for environmental protection and resource depletion, sustainable green development has become an everlasting international topic and our pursuit of sustainable green materials has never stopped. Therefore, the use of natural biodegradable materials such as cellulose [1], chitin [2] and starch [3] has been greatly promoted.

In recent years, a nanocellulose [4,5] with excellent properties such as adjustable morphology, high specific surface area, high aspect ratio, biodegradability, biocompatibility, surface chemical reactivity, and high mechanical properties [6,7] has attracted the interest of many researchers, and it can be applied in biomedicine, food packaging, electronic devices, energy storage materials, diaphragms, optical materials, coatings, reinforced toughening materials and water purification [8,9,10].

Waste paper, as a potential and easily neglected source of nanocellulose [11], requires no excessive physical and chemical treatment to remove lignin and hemicellulose because most of the lignin and hemicellulose in lignocellulosic biomass is already removed by the papermaking process [12,13,14]. Lignin and hemicellulose are removed using sodium hydroxide and sodium sulfite at 80–90 °C, followed by bleaching by sodium hypochlorite to further remove lignin. After recycling, waste paper is generally used to produce low-value recycled paper. Waste paper contains a high cellulose content (60–70%) because of the debinding and bleaching process, while the total amount of hemicellulose and lignin is often less than 25% [11]. This is an irresistible temptation for the preparation of high value-added nanocellulose.

Prior to the preparation of nanocellulose, waste paper is usually pretreated to remove impurities (e.g., lignin, hemicellulose, and ink) other than cellulose. As shown in Table S1, the alkali treatment is the most common method of pretreatment to remove lignin and ink from waste paper [15,16,17,18,19,20,21,22]. In this paper, the D_0_E_P_D_1_ sequential bleaching process [23,24] was used, which can selectively remove lignin due to the low oxidation potential of ClO_2_, resulting in high bleaching efficiency, a high yield of the obtained bleached pulp, good whiteness stability, anticorrosive and bactericidal effects [25]. Meanwhile, it can reduce the acid value, toxicity, color, sodium chloride and organic halide content of bleached wastewater, and reduce the cost and water consumption of treated wastewater. The E_P_ stage is mainly for further removal of degraded lignin in the D_0_ stage [26], while hydrogen peroxide as a green oxidation promoter can not only prevent serious degradation of cellulose in the E_P_ stage, but also reduce the use of ClO_2_ in the D_0_ stage. The D_1_ stage [27] is generally used to increase whiteness.

Nanocellulose is usually classified into cellulose nanocrystal (CNC) [28], cellulose nanofiber (CNF) [29], and bacterial cellulose (BC) [30]. At present, for the preparation of nanocellulose from waste paper, only a simple acid hydrolysis method [31,32,33] is available. This method requires large amounts of sulfuric acid because the amorphous regions of cellulose can only be hydrolyzed by acid without complete hydrolysis to carbon dioxide and water when a high concentration of sulfuric acid is maintained. In subsequent operations, it requires ten times more water than the reaction solution to terminate the reaction. At the same time a large amount of water is used for the dialysis process, producing a large amount of acidic wastewater. This is very detrimental to the current increasingly tight competition for water resources.

Therefore, we adopted the more gentle and industrial-friendly TEMPO-oxidation method [34,35,36]. Isogai et al. described in detail the mechanism by which TEMPO selectively oxidizes the primary hydroxyl groups on cellulose to aldehyde groups and finally to carboxyl groups [37]. They also clarified that TEMPO oxidation of cellulose can make a significant contribution in terms of sustainable societies and it is environmentally friendly. In this paper, the TEMPO/NaBr/NaClO system at pH 10–11 at room temperature was adopted. In addition, the total amount of office waste paper is relatively large but the utilization rate is the lowest [11]. The three different pretreatments of acid, alkali, and bleaching were applied for office waste paper as the research object to discuss the effects of different pretreatment methods on the quality of the produced CNFs and to obtain a relatively green and environment-friendly method for mass production of the CNFs.

## 2. Materials and Methods

### 2.1. Material

The office waste paper used in this work comes from discarded paper documents and was cut into 0.5 × 1 cm^2^ size by a shredder. Subsequently, it was crushed with a household high speed blender for 15 min to prepare 10 wt% waste paper (WP) pulp. Bleached pulp board (BPB) (Dalian Yangrun Trading Company, Dalian, China), Chlorine dioxide (ClO_2_) (Guangdong Institute of Microbiology, Guangzhou, China), 2,2,6,6-tetramethyl-piperidine-1-oxyl (TEMPO) (Shanghai Macklin Biochemical Co., Ltd., Shanghai, China). The sulfuric acid (H_2_SO_4_), sodium hydroxide (NaOH), sodium hypochlorite (NaClO), sodium bromide (NaBr), and hydrogen peroxide (H_2_O_2_) were purchased from Damao Chemical Reagent Factory (Tianjin, China).

### 2.2. Low Acid Treatment

WP were treated with 1 wt% sulfuric acid solution under reflux at 135 °C for 30 min using a WP/H_2_SO_4_ solution ratio of 1:20 (*w*/*v*). Afterward, the solutions were filtered and washed with deionized water until neutral, and dried for 24 h in an oven with air circulation at 80 °C, labeled as WP1.

### 2.3. Alkali Treatment

WP were treated with 12 wt% NaOH solution under reflux at 90 °C for 120 min using a WP/NaOH solution ratio of 1:20 (*w*/*v*). Afterward, the solutions were filtered and washed with deionized water until a neutral, and dried for 24 h in an oven with air circulation at 80 °C, labeled as WP2.

### 2.4. Bleaching Treatment

WP, WP1 and WP2 was bleached by the D_0_(E_P_)D_1_ bleaching sequence (where D represents chlorine dioxide stage and (E_P_) represents the hydrogen peroxide-reinforced alkaline extraction stage), labeled as BWP, BWP1, BWP2. Firstly, the content of waste paper and ClO_2_ in the reaction solution in the D_0_ stage was 10 wt% and 2 wt%, respectively. The pH of the reaction solution is adjusted to 2.5 by sulfuric acid and the waste paper is reacted at 70 °C for 45 min. Then, in the (E_P_) stage, 2 wt% NaOH and 0.5 wt% H_2_O_2_ are added to 10 wt% waste paper pulp and the reaction is carried out at 70 °C for 60 min. In the final D_1_ stage, 0.5 wt% ClO_2_ was added to the 10 wt% waste pulp and the final pH of the (D_1_) stage was adjusted to 4 with 1 M NaOH solution and the waste paper was reacted at 70 °C for 60 min. Afterward, the solutions were filtered and washed with deionized water until neutral, and dried for 24 h in an oven with air circulation at 80 °C.

### 2.5. Preparation of Cellulose Nanofibers

All office waste paper fiber samples and bleached pulp boards were prepared into CNFs by the TEMPO/NaBr/NaClO system at pH 10. It required 100 mL of deionized water per gram of a sample dispersed overnight, then the addition of 0.1 mmol TEMPO mixed with 1 mmol NaBr at room temperature and stirred. We waited for the complete dissolution of TEMPO, then added a 20 mmol NaClO solution, and a 0.5 M NaOH solution was used to maintain the pH of the mixture within a range of 10–10.5 during the reaction. When the pH was completely stabilized at 10, we added an excess of anhydrous ethanol to end the reaction, and the resulting jelly clear product was obtained by vacuum filtration, placed in a flask, and then 100 mL of deionized water was added and it sonicated for 1 h to obtain a homogeneous, light blue CNFs solution. The TEMPO-oxidized CNFs prepared by WP, WP1, WP2, BWP, BWP1, BWP2, and BPB were named WCNF, WCNF1, WCNF2, BCNF, BCNF1, BCNF2, and PCNF, respectively.

### 2.6. Characterization

#### 2.6.1. Morphology

The morphology of each fiber was observed with a SU8030 field emission scanning electron microscope (FE-SEM) (Hitachi High Tech Co., Ltd., Tokyo, Japan). The analysis was performed at an accelerating voltage of 5 kV. The samples were coated with a layer of platinum prior to analysis to prevent overcharging. The particle size of the nanocellulose was further observed with a JEM 2100 high-resolution transmission electron microscope (Hitachi High Tech Co., Ltd., Tokyo, Japan) at an accelerating voltage of 200 kV. The diluted CNFs suspension was sonicated for 15 min and then dropped onto a copper grid of copper foil for freeze-drying.

#### 2.6.2. Particle Size Distribution and Zeta Potential

The TEMPO-oxidized CNFs solution was ultrasonically dispersed for 15 min, and then put into a transparent quartz tube, and a Submicron Particle Size and Zeta Potential Analyzer (Beckman Coulter, Los Angeles, CA, USA) was used to measure the particle size distribution. The above solution was prepared as a 0.5 wt% CNFs solution (pH adjusted to 7–8) and a small amount of the solution was injected into the measurement device using a syringe to test the zeta potential. The instrument uses photon correlation spectroscopy and electrophoretic light scattering technology to measure the particle size and Zeta potential of the particles in the suspension. The suspension can be directly detected without dilution.

#### 2.6.3. Fourier Transform-Infrared (FTIR)

FTIR analysis was performed on each sample to follow the changes in chemical composition and functional groups of all fibers before and after TEMPO oxidation. A total of 100 mL of 0.5 wt% CNFs solution was prepared and placed in Petri dishes and dried into films in an oven at 60 °C. The infrared spectra of the samples were analyzed with an iS50R FTIR spectrometer (Thermo Fisher Scientific, Waltham, MA, USA) with a resolution of 4 cm^−1^ and a scan rate of 32 scans per minute with wavenumbers ranging from 4000 to 400 cm^−1^. Before analysis, the samples without TEMPO oxidation were first ground and mixed with 1:10 (*w*/*w*) KBr powder and pressed into small ultra-thin slices. The functional groups in the samples were detected by absorbance curves of FTIR spectra. All TEMPO-oxidized CNFs samples were analyzed by iS50R Fourier transform infrared spectroscopy (Thermo Fisher Scientific, Waltham, MA, USA) using the attenuated total reflection (ATR) technique.

#### 2.6.4. Optical Performance

The transmission spectra were obtained with a UV-Vis NIR spectrophotometer (SHIMADZU, Shanghai, China) in the visible wavelength range (800–400 nm). The sample was placed in front of the input window of the integrating sphere and the total transmission (*T_t_*) (direct and diffuse transmission), and direct transmission (*T_d_*) were measured. In order to quantify and compare the diffusive optical transmission properties of films, we define the haze parameter χ, using the following Equation (1):

(1) $$\chi =\left(T_{t}-T_{d}\right)/T_{t}\times 100\%$$

#### 2.6.5. X-ray Diffraction (XRD)

The effect of pretreatment on the crystallinity index (CrI) [15,38] of the nanocellulose samples can be assessed from the generated X-ray diffractograms. X-ray diffraction analysis (XRD) was performed using an Ultima IV diffractometer (Rigaku, Tokyo, Japan) to determine the relative amounts of crystalline phases in samples of nanocellulose films prepared from office waste paper fibers. The nanocellulose films were placed on a sample holder and scanned in the range of 2θ = 3° to 60°. The crystallinity index indicates the relative crystallinity, and the diffraction intensity data of TEMPO-oxidized CNFs were measured by Segal’s empirical method, using the following equation [39]:

(2) $$\mathrm{CrI}=\left(I_{002}-I_{am}\right)/I_{002}\times 100\%$$

where *I*_002_ represents the maximum peak intensity of the lattice diffraction (002) in the crystalline region (2θ = 22.5°) and *I_am_* is the minimum between the planar reflection (002) and (110), referring to the reflection intensity of the amorphous part of the XRD spectrum (2θ = 18.5°).

#### 2.6.6. Thermogravimetric Analysis (TGA)

The thermal stability of all TEMPO-oxidized CNFs samples was investigated using a TGA2 thermal analyzer (METTLER TOLEDO, Shanghai, China) for thermogravimetric analysis. The sample ramp-up rate was 10 °C/min, the ramp-up temperature range was 30–600 °C, the sample weight was 6 ± 0.5 mg, the crucible material was alumina, and the protective atmosphere was nitrogen. The decomposition temperatures of all TEMPO-oxidized CNFs samples with weight loss were determined based on the changes in weight loss curves and the differential heat curves of thermal analysis.

#### 2.6.7. Yields

TEMPO-oxidation yield was calculated by Equation (3):

(3) $$\mathrm{TEMPO}\;\mathrm{oxidation}\;\mathrm{yield}\;\left(\%\right)=\left(m_{CNF}/m_{a}\right)\times 100$$

where *m_CNF_* is the dry mass of the produced *CNF*s (with the remaining impurities) and *m_a_* is the dry mass of sample after pretreatment.

The *CNF*s of the process yield was determined by the ratio of *m_CNF_* and the initial dry mass before pretreatment, as shown in Equation (4):

(4) $$\mathrm{process}\;\mathrm{yield}\;\left(\%\right)=\left(m_{CNF}/m_{b}\right)\times 100$$

where *m_b_* is the initial dry mass before pretreatment.

## 3. Results

### 3.1. Morphology Analysis

**Figure 1:**
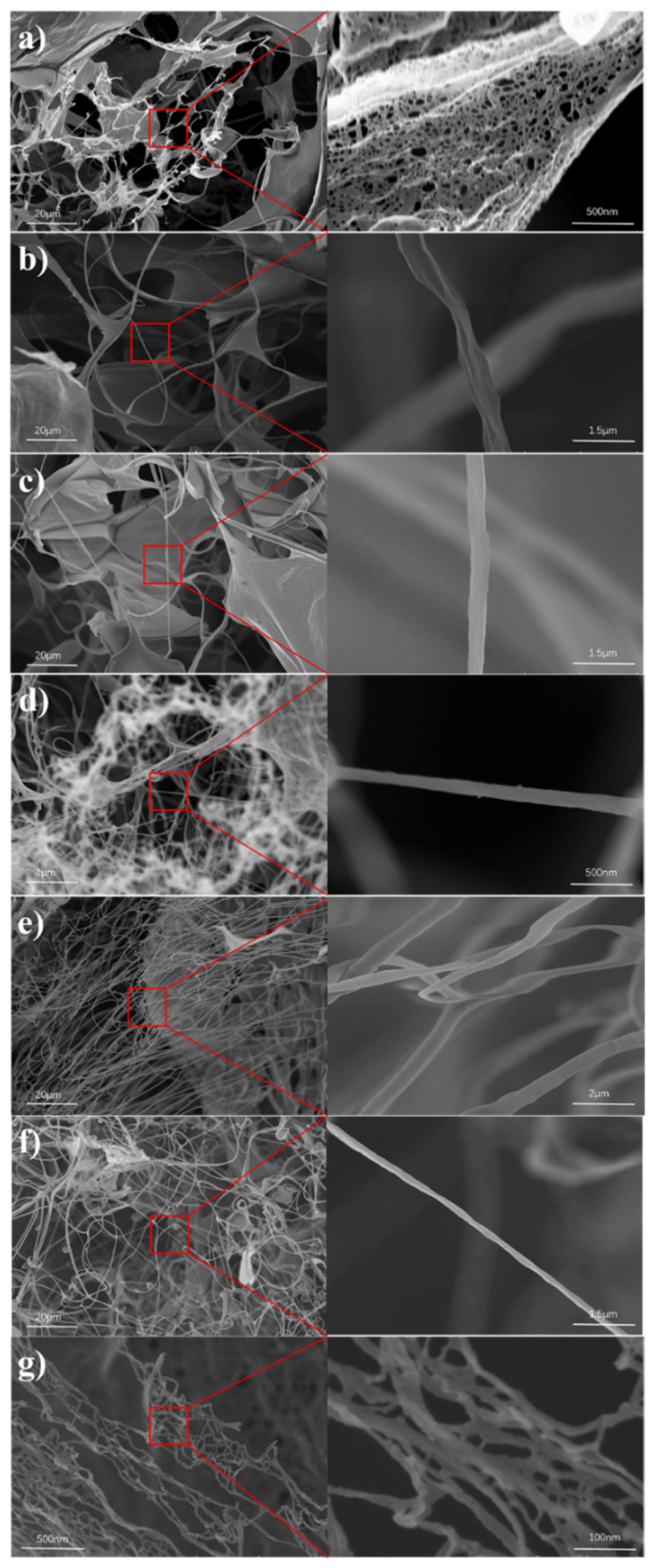
SEM images of the CNFs. (**a**) WCNF, (**b**) WCNF1, (**c**) WCNF2, (**d**) BCNF, (**e**) BCNF1, (**f**) BCNF2, (**g**) PCNF.

The fiber morphology of all TEMPO-oxidized CNFs samples was observed by SEM. Figure 1a shows a CNFs sample prepared from office waste paper fibers without any pretreatment, and it can be clearly seen that the WCNF takes a fiber-sheet shape after freeze-drying. In the partial enlarged image on the right, there are many nanopore structures on the leaf surface due to cellulose accumulation, which indicates that office waste paper containing high cellulose content can be prepared into CNFs without any pretreatment. Figure 1b,c show the CNFs obtained from acid-treated and alkali-treated office waste paper fibers, respectively. They possess a structure similar to that of the WCNF, and it can be seen that the individual large fibers have a diameter of about 1 μm or less, and form a dense flaky surface due to the tight accumulation of cellulose. It was also found that ink particles were visible on the lamellar surface formed by the acid-treated WCNF1 sample, while the lamellar surface of the alkali-treated WCNF2 sample was very smooth without ink particles remaining, as seen in Figure S1.

Surprisingly, the more delicate fiber network can be clearly seen in all bleached samples, indicating that the bleaching pretreatment is beneficial to further disperse the office waste paper fibers. The local enlarged images on the right side show BCNF, BCNF1, and BCNF2, respectively, with diameters of about 500 nm or less. Moreover, the fibers of BCNF and BCNF2 are be finer compared with BCNF1. In addition, we found that the BCNF1 samples have the potential to prepare CNFs with high aspect ratio, and long fibers with lengths longer than 0.5 mm and widths less than 0.5 μm can be clearly seen under SEM, as shown in Figure S2. Among all TEMPO-oxidized CNFs samples, the CNFs prepared by BPB have higher quality as a control group, Figure 1g showing its diameter in the range of 10 to 20 nm. This is because the pure bleached pulp board without remaining impurities, the excess lignin and hemicellulose are removed during the TEMPO-oxidation process, and most of the intercellulose hydroxyl groups can be converted into aldehyde and carboxyl groups, which is more desirable for CNFs samples.

**Figure 2:**
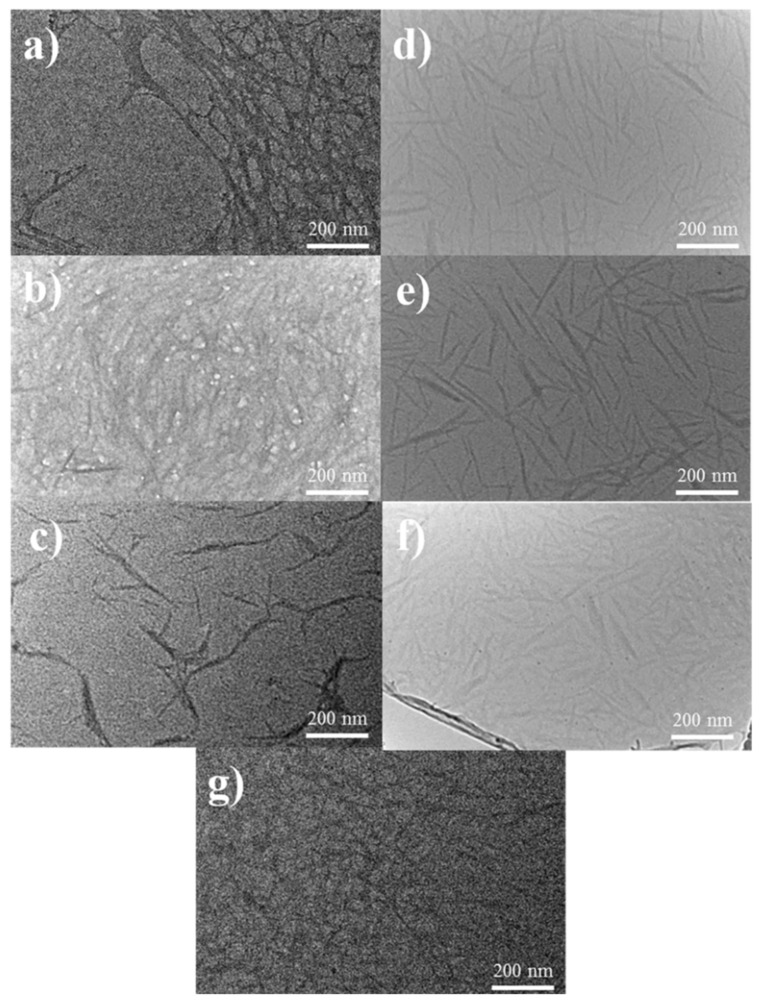
TEM images of the CNFs. (**a**) WCNF, (**b**) WCNF1, (**c**) WCNF2, (**d**) BCNF, (**e**) BCNF1, (**f**) BCNF2, (**g**) PCNF.

TEM characterization was also carried out to further investigate the morphology structure of the TEMPO-oxidized CNFs. Figure 2 show the TEM profile of TEMPO-oxidized CNFs with different pretreatment methods and materials. We observed by TEM measurements that office waste paper and bleached pulp board fibers successfully fibrillated from micron size to nanoscale. Theoretically, the width of the nanocellulose should be constant [40], but we can clearly observe that all TEMPO-oxidized CNFs have diameters between 5 and 20 nm. The lengths are widely distributed and varied depending on the materials, oxidation and disintegration conditions.

The TEM images of WCNF formed an entangled, complex spider-like geometry, indicating that the unprepared office waste paper fibers did not completely disintegrate during the TEMPO-oxidation process and that some of the fiber network was in an unfibrillated state. WCNF1 showed a thread-like shape with fibers all piled up together, and WCNF2 had a longer diameter and larger width among all TEMPO-oxidized CNFs samples, probably due to agglomeration during the preparation process.

Interestingly, the bleached pretreated office waste paper fibers after TEMPO oxidation showed needle-like morphology, which was very similar to that of CNC. However, BCNF, BCNF1 and BCNF2 possessed a dense continuous fiber network rather than a needle-like fiber stacking structure in the above SEM images and did not satisfy the high crystallinity of CNC in the subsequent XRD analysis, so the nanocellulose prepared from bleached treated waste paper fibers remains classified as CNFs. It is possible that the secondary bleaching treatment caused the waste paper fibers to break, and these broken cellulose reacted more easily with the TEMPO/NaBr/NaClO system, making the prepared TEMPO-oxidized CNFs length smaller.

The TEMPO-oxidized CNFs prepared from BPB without any pretreatment exhibited a well-dispersed fiber network due to the presence of a large number of negatively charged sodium carboxylate groups on the surface of TEMPO-oxidized CNFs, which may lead to the formation of such sufficiently individualized long cellulose nanofibers through electrostatic repulsion and/or permeation effects in water [37].

### 3.2. Particle Size Distribution and Zeta Potential

**Figure 3:**
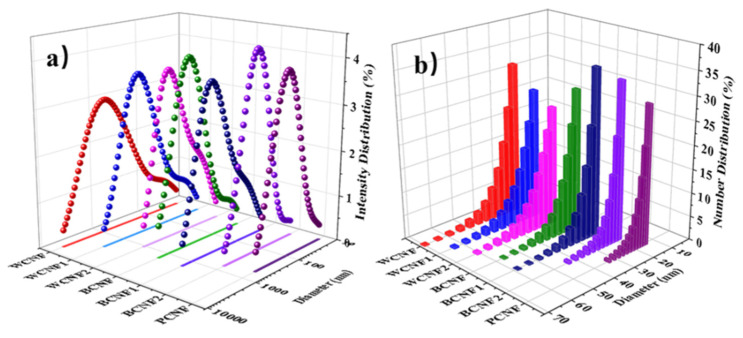
Particle size distribution of the CNFs. (**a**) intensity-size distribution, (**b**) number-size distribution.

The laser particle sizer is a precision instrument that measures the size of powder particles based on the principle of light scattering. It is not accurate for measuring the size of fibers, but it can infer the overall distribution of the prepared CNFs from the measured intensity-size distribution data. The BCNF1, BCNF2 and PCNF in Figure 3a show pleasing normal distributions, with very sharp peaks for BCNF2 and PCNF, indicating a relatively concentrated nanocellulose scale distribution with intensity-size distributions of roughly 52.8–331.1 nm and 37.3–264.5 nm, respectively. However, the peaks of WCNF, WCNF1, and BCNF are relatively low, and the bottom projection shows a wide intensity-size distribution and a buffer platform in the area of less than 100 nm, which may be caused by residual ink particles. The intensity-size distribution of WCNF2 (52.9–577.9 nm) was second only to BCNF2, indicating that the alkali-treated office waste paper was beneficial for the preparation of nanocellulose with concentrated scale distribution. In conclusion, the CNFs obtained from office waste paper without any pretreatment had the worst intensity-size distribution, while the size distribution of CNFs obtained from office waste paper fibers with two pretreatments (alkali treatment and bleaching treatment) is very close to that of PCNF. The particle size distribution of all TEMPO-oxidized CNFs can be found in Table 1.

To further understand the scale distribution of CNFs, we also formed number-size distribution plots of all TEMPO-oxidized CNFs, as shown in Figure 3b. We can obtain the average diameter by calculated number size distribution as shown in Table 1. The obtained average diameters are also very close to the diameters of CNFs on TEM images. The results are clear that the average diameter of the bleached CNFs samples is less than 26.01 nm. However, the average diameters of WCNF1 and WCNF2 are 30.62 nm and 30.27 nm, respectively, which means that the bleaching treatment is favorable to improve the fibrillation of office waste paper.

Due to electrostatic repulsion of anionic carboxylate groups on the surface of the cellulose after TEMPO oxidation, the CNFs were able to disperse uniformly in the aqueous solution. As shown in Table 1, the zeta potential of all pretreated CNFs samples was less than −29.58 mV, which indicates that the pretreatment made the CNFs prepared from waste paper have good dispersion stability. It is worth mentioning that the zeta potentials of BCNF and BCNF1 were −37.83 and −41.92 mV, respectively, which were much higher than those of WCNF (zeta potential of −19.01 mV), and to some extent this indicates that the bleaching treatment helped to improve the dispersion stability.

### 3.3. FTIR

**Figure 4:**
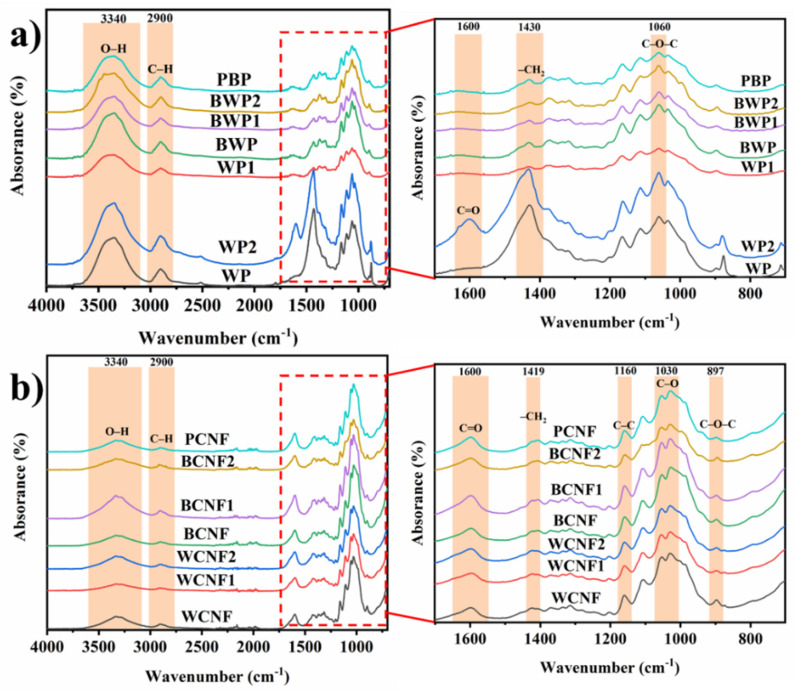
FTIR spectra of all samples before and after TEMPO oxidation. (**a**) Fiber sample before TEMPO oxidation, (**b**) CNFs sample after TEMPO oxidation.

The changes in chemical functional groups of different samples before and after TEMPO-oxidation catalysis were analyzed by FTIR spectra. Figure 4a shows the FTIR spectra of all samples before TEMPO oxidation, including unpretreated and pretreated office waste paper, and bleached pulp board. Figure 4b shows the FTIR spectra of different samples after TEMPO oxidation. It can be clearly seen that the characteristic intervals of both images are mainly in the high wavenumber region (3650–2800 cm^−1^) and the low wavenumber region (1700–500 cm^−1^). In the high wavenumber region, a broad band is clearly seen between 3650 and 3000 cm^−1^, which is related to the free O-H stretching vibration of the hydroxyl group in the cellulose molecule at 3340 cm^−1^. Meanwhile, the peak at 3650–3000 cm^−1^ becomes weaker after oxidation of all samples in the TEMPO/NaBr/NaClO system, which is due to the fact that the primary hydroxyl group between the cellulose molecules were oxidized to aldehyde and carboxyl groups by TEMPO. Due to the asymmetric and symmetric stretching vibrations of -CH_2_, -CH and hydroxyl groups in the aliphatic bonds of lignin, hemicellulose and cellulose (about 2900 cm^−1^), peaks were detected in the region of 3000–2800 cm^−1^, and undoubtedly, the peaks here also weakened with TEMPO oxidation.

In Figure 4a, a strong peak was detected at 1600 cm^−1^ for the FTIR spectrum of WP2, which was attributed to the stretching vibration of the C=O bond on the carboxyl group, indicating that some components of the office waste paper formed carboxylate groups after saponification. Compared with WP2, FTIR images of all CNFs samples formed a new peak at 1600 cm^−1^, as shown in Figure 4b, corresponding to the C=O bond stretching vibration [41] from the carboxylate carboxyl group (-COO-). This indicates that TEMPO-oxidized the hydroxyl groups on cellulose C6, leading to a significant increase in the number of aldehyde and carboxyl groups. The band at 1030 cm^−1^ corresponds to the stretching of the C-O and C-C bonds [42] compared with the sample before TEMPO oxidation, and the very intense absorption peak indicates the presence of a large amount of cellulose, which is due to the substantial increase in the proportion of cellulose as most of the impurities were removed during the oxidation process. The small peak around 897 cm^−1^ in Figure 4b, representing the glycosidic C1-O-C4 deformation characteristic of the β-glycosidic link in cellulose [35], appeared weaker for all CNFs samples, which indicated a scission of the β-glycosidic bond by chemical treatments. The increase of the intensity of the peak in the region of 1055 cm^−1^ were associated with the C-O-C pyranose ring [38,43] (antisymmetric in phase ring) stretching vibration, which demonstrates the efficiency of the removal of lignin and non-cellulose components from the office waste papers in the pretreatments. Furthermore, the C-C ring breathing band at about 1160 cm^−1^ arose from the polysaccharide component [38]. The absorption band detected in the region of 1200–1450 cm^−1^ is related to the bending vibration of the CH_2_OH group [36] on the C6 of cellulose. Comparing Figure 4a,b, the symmetrical CH_2_ bending at 1430 cm^−1^ was weakened and transferred to 1419 cm^−1^ after TEMPO oxidation, proving the formation of new hydrogen bonds [44].

### 3.4. Optical Performance

**Figure 5:**
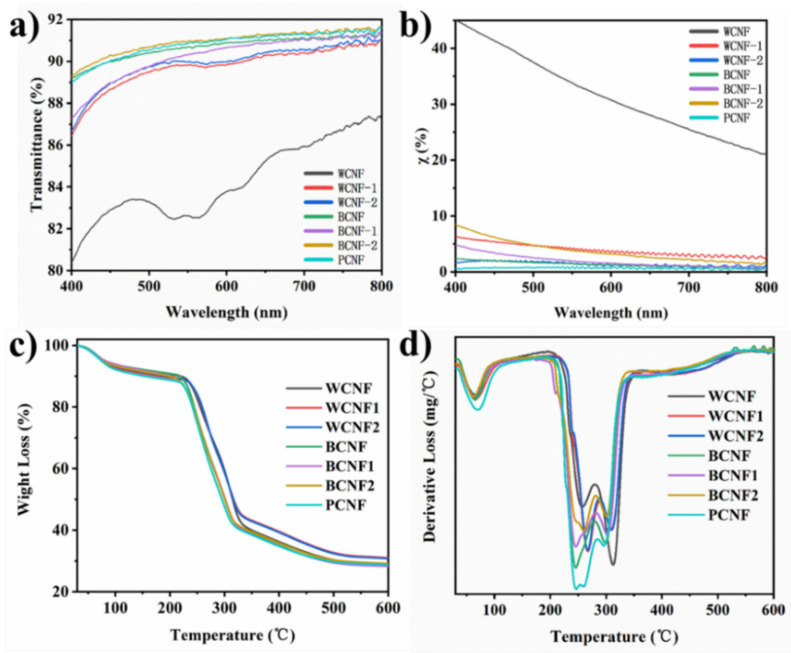
The optical properties and thermal stability of different TEMPO-oxidized CNFs films. (**a**) The transmittance, (**b**) the diffusive optical transmission properties, (**c**) TG and (**d**) DTG curves.

We investigated the optical properties of all TEMPO-oxidized CNFs films. Figure 5a shows the total transmission of visible light measured by integrating the sphere mode, which shows that the transmittance of pretreated office waste paper is basically above 86.5%, and the transmittance of bleached office waste paper is higher than that of the unbleached sample in the range of 600–800 nm. Meanwhile, the transmittance of BCNF and BCNF2 was almost the same as that of PCNF, indicating that the bleaching treatment had a certain effect on improving the visible transparency. The WCNF films showed the worst light transmission under the same nano-fibrillation technique, while the other pretreated CNFs films showed similar light transmission as the PCNF films, indicating that some degree of pretreatment helps to increase the visible light transmission.

Figure 5b shows the diffusive optical transmission properties of the different CNFs films in the visible region. As shown in Figure 5b, all the CNFs films show a trend of decreasing the scattering transmission parameter χ with increasing wavelength [45]. Among them, the χ of the WCNF films was much higher than that of the rest of the CNFs films, and such a high χ in the near-UV region made the films with the solution also appear purple instead of the light blue color of the other film samples (Figure S3). It is worth mentioning that the CNFs solution remained stable at room temperature after 66 days. The χ value of PCNF was the lowest, and the alkali-treated WCNF2 and the bleached-only BCNF were also the closest to it. In contrast, the average χ values of BCNF2 and WCNF1 were six times higher than those of PCNF, indicating that acid treatment and excessive pretreatment increased the scattering transmission of the CNFs films, as shown in Table 2. In general, pretreatment is important to improve the optical properties of the CNFs films, and the optical properties of only the alkali-treated CNFs films are closest to those of the CNFs films prepared from bleached slurry plates.

### 3.5. Thermal Stability

The thermal stability of seven TEMPO-oxidized CNFs was determined by the thermogravimetric method. It is essential to understand the thermal degradation properties of the materials for their potential use in industrial applications. It is well-known that the thermal stability of polymeric materials depends on the intrinsic properties of the samples and the molecular interactions between different macromolecules. In Figure 5c, we can see that all the TEMPO-oxidized CNFs have a large mass loss between the region of 30–110 °C. Combined with the DTG curve shown in Figure 5d, a clear peak can also be seen where the first mass loss of the TEMPO-oxidized CNFs films is mainly the evaporation or/and removal of its own moisture during the warming process. This is attributed to the inherent hydrophilic and hygroscopic properties of nanocellulose, where the CNFs film samples absorb moisture from the surrounding environment during the preparation and preservation stages, and the TEMPO-oxidized CNFs films themselves possess adsorbed water. In addition, this may also be related to the decomposition of the low molecular compounds attached to the films [46].

The DTG curve [47] shows two decomposition peaks in the range of 200–360 °C for the TEMPO-oxidized CNFs films, and this area is also consistent with the main mass loss region of the TG curve. The decomposition peaks in the temperature range of 246–290 °C were mainly caused by the breakage of cellulose glycosidic bonds and the thermal depolymerization of the residual hemicellulose, which was consistent with the glycosidic bonds detected by the FTIR spectroscopy at 897 cm^−1^. The decomposition peaks located in the temperature region of 290–313 °C are mainly due to the depolymerization of the cellulose structure. In general, when the temperature reaches 360 °C, all cellulose basically decomposes. In contrast to cellulose, hemicellulose consists mainly of amorphous structures and it is the first compound to be decomposed during the warming process. Whereas the structure of lignin carries a larger number of different aromatic rings; therefore, the decomposition temperature range of lignin is very wide and can sustain from 200 °C to 600 °C. The different chemical structures of cellulose, hemicellulose and lignin lead to different thermodynamic differences.

According to the IOS standard, the two points of 20% and 50% weight loss are identified on the TG curve, and the point obtained by intersecting the line where the two points are located with the baseline extension line is the decomposition temperature. As shown in Table 3, the decomposition temperature of WCNF was 214.7 °C, which was slightly lower than that of WCNF1 and WCNF2 (217.6 °C and 218.8 °C, respectively). However, the decomposition temperatures of the CNFs samples prepared from bleached waste paper fibers ranged from 201 to 205 °C, which was similar to the decomposition temperature of PCNF (204 °C). WCNF1 and WCNF2 had the best thermal stability and BCNF, BCNF1 and BCNF2 had the poorest thermal stability considering three factors affecting the thermal stability such as decomposition temperature, the decomposition peak of the DTG curve and the residual rate. This indicates that less-acid treatment and alkali treatment contribute to improve the thermal stability of CNFs, while bleaching treatment decreases their thermal stability. The thermal stability of the CNFs is associated with an increase in crystallinity, and fibril and particle size. Because the high crystallinity with a larger specific surface area provides the CNFs a better heat transfer capacity, it causes the degradation of the surrounding fibers when one fiber is exposed to a heat source and starts to degrade. In addition, the smaller particle size can have more free chains, which decomposes at lower temperatures, resulting in more char. Meanwhile, the presence of inorganic salts and acids can act as a flame retardant, resulting in an increased char yield, so that the carbon residuals of WCNF1 and WCNF2 are higher.

### 3.6. Crystallinity

**Figure 6:**
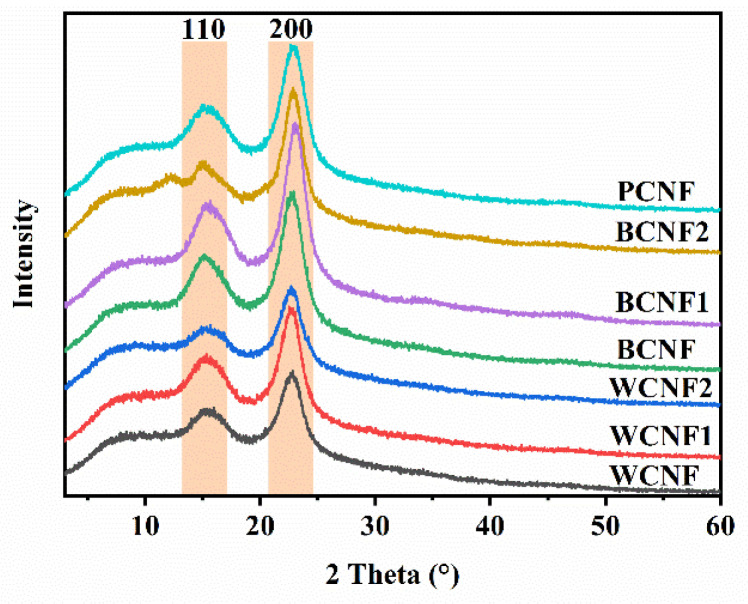
X-ray diffraction patterns of TEMPO-oxidized CNFs.

The TEMPO-oxidized CNFs were further characterized by X-ray diffractometry to examine their chemical properties and crystallinity index. The crystallinity index is an important characteristic that affects the mechanical strength in a polymer matrix. As can be seen from Figure 6, the obtained CNFs samples have a diffraction pattern similar to that of natural cellulose, with diffraction peaks located at 2θ = 15° and 22.5°, corresponding to the (110) and (200) lattice planes, respectively, which belong to the typical cellulose I structure. As it can be seen from Table 2, the crystallinity of the WCNF1 sample was as high as 60.24%, which was 10% higher than that of the office waste paper fibers without any treatment, while the crystallinity of the WCNF2 sample was slightly lower than that of WCNF, due to the fact that the amorphous region in office waste paper fibers is more easily hydrolyzed under acidic conditions, resulting in the exposure of hydrogen bonds between cellulose molecules, which are subsequently the TEMPO-oxidation process gradually transformed into sodium C6–carboxylate groups.

Surprisingly, the BCNF after bleaching pretreatment had the highest crystallinity (62.22%) among the three pretreatments, which was 13.5% higher than WCNF, which had surpassed PCNF (61.47%), probably because the hydrogen peroxide removed more amorphous regions and excess lignin during the bleaching process, while the interior of the crystalline region remained intact and the TEMPO-oxidation process can only take place on the cellulose surface, and therefore possesses a higher crystallinity. In addition, the acid-treated and alkali-treated samples after bleaching treatment also obtained satisfactory results, with a crystallinity of 66.41% and 65.27%, respectively. Combined with the needle-like CNFs observed in the TEM image (similar to the highly crystalline CNC), it is also further verified that the bleach-treated CNFs samples have high crystallinity.

The above results show that acid treatment and bleaching treatment can effectively improve the crystallinity of the CNFs, and the TEMPO-oxidized CNFs obtained by acid treatment before bleaching treatment has the highest crystallinity, i.e., BCNF2 has better mechanical properties and tensile strength, which is beneficial to the application of composite materials.

### 3.7. Yields

In the current study, two calculations were adopted to represent the sample yields before and after pretreatment, as shown in Table 2, where 61.9% and 72.5% yields were obtained for the CNFs prepared from office waste paper and bleached pulp board without any pretreatment, respectively. From the results obtained above, the quality of WCNF was substandard, while PCNF, which was used as a control, performed the best. In addition, the yield of acid-treated WCNF was 25% higher than that of alkali-treated WCNF, which can be due to the long alkali treatment process, resulting in some components (e.g., ink, filler) being decomposed into small molecules during the reaction and removed with the extraction process. The process yields of bleached BCNF and BCNF1 (40.58% and 39.98%, respectively) were not high, but the TEMPO yields (60.88% and 62.02%, respectively) were close to those of WCNF. However, in the above test results, the yield of BCNF2, which has a relatively good performance, was very low, which can be due to the excessive pretreatment process flow, and therefore the too much loss in the material transfer and extraction process during the experimental process, which is very unfavorable for industrialization. In summary, BCNF has a small diameter, narrow particle size distribution, excellent optical properties, high crystallinity and yield, and comparable thermal stability, which is suitable for industrial production of office waste paper.

## 4. Discussion

We subjected office waste paper to three different pretreatment processes, namely acid treatment, alkali treatment and D_0_E_P_D_1_ sequential bleaching treatment, to investigate the effect of different pretreatment processes on the prepared CNFs. Few experiments have been conducted specifically for office waste paper bleaching, and we adopted a greener bleaching method, which is important in terms of energy saving and emission reduction. In addition, we employed the TEMPO/NaBr/NaClO system to prepare the CNFs, which has the potential to be scaled up and reduce energy consumption on a large scale.

The experimental results of SEM and TEM showed that all samples were successfully nano-fibrillated by TEMPO oxidation, which confirmed the feasibility of the TEMPO-oxidation method for the preparation of the CNFs from office waste paper. In addition, the results of particle size distribution and zeta potential indicated that the bleached CNFs solution had a narrow particle size distribution, a small average diameter and good dispersion stability. The FTIR spectra also confirmed that the intermolecular C6 hydrogen bonds were oxidized to carboxyl and aldehyde groups during the TEMPO-oxidation process. The UV spectra illustrated that the CNFs films with bleaching treatment had good transmittance in the visible range. XRD and TG confirmed the high crystallinity and poor thermal stability of the bleached CNFs compared with other CNFs samples prepared from office waste paper. Finally, the yield is an important reference factor for practical applications; BCNF and BCNF1 have relatively high yields (40.58% and 39.98%, respectively) from the office waste paper to the preparation into the CNFs.

## 5. Conclusions

In summary, bleaching treatment is beneficial to improve the quality and yield of CNFs made from office waste paper. Meanwhile, considering the practical production application, it is a good choice to produce BCNF with suitable particle size distribution, environment-friendly pretreatment and scalable production.

## Figures and Tables

**Table 1:** Particle size distribution and zeta potential of the CNFs.

Sample	D_10_^1^ (nm)	D_50_^2^ (nm)	D_90_^3^ (nm)	Average Dimension (nm)	Zeta Potential (mV)
WCNF	80.5	583.1	2363.1	31.92	−19.01
WCNF1	66.3	354.4	1100.7	30.62	−32.00
WCNF2	52.9	217.7	577.9	30.27	−31.20
BCNF	87.4	317	873.2	26.01	−37.83
BCNF1	63.1	292.1	822.3	25.07	−41.92
BCNF2	52.8	135.7	331.1	21.40	−29.58
PCNF	37.3	104.8	264.5	17.10	−40.01

^1^ D_10_, ^2^ D_50_ and ^3^ D_90_ represent the particle size corresponding to a sample with a cumulative particle size distribution of 10%, 50% and 90%, respectively. Its physical meaning is that particles smaller than it account for 10%, 50% and 90% of the particle size, respectively.

**Table 2:** Scattering transmission parameter, crystallinity index and yield data of the CNFs.

Samples	Average χ (%)	CrI (%)	Process Yield (%)	TEMPO-Oxidation Yield (%)
WCNF	31.63	54.80	61.90	61.90
WCNF1	3.92	60.24	43.25	48.63
WCNF2	1.38	54.62	34.70	39.70
BCNF	1.33	62.22	40.58	60.88
BCNF1	1.91	66.41	39.98	62.02
BCNF2	3.68	65.27	21.61	41.97
PCNF	0.66	61.47	72.50	72.50

**Table 3:** Thermal data from TG analysis of the CNFs.

Samples	T_a_^1^ (°C)	T_b_^2^ (°C)	Decomposition Temperatures (°C)	Residual Rate (%)
WCNF	257.3	312.8	214.7	28.57
WCNF1	267.0	309.8	217.6	30.97
WCNF2	268.0	310.0	218.8	30.72
BCNF	246.0	299.0	205.0	28.50
BCNF1	245.8	302.0	201.0	28.27
BCNF2	260.0	300.8	203.3	29.20
PCNF	246.6	296.6	204.0	28.73

T_a_^1^: Maximum weight loss temperature of the first decomposition stage. T_b_^2^: Maximum weight loss temperature of the second decomposition stage.

## Data Availability

The data that support the findings of this study are available from the corresponding author upon reasonable request.

## Supplementary Materials

The following are available online at https://www.mdpi.com/article/10.3390/polym13183119/s1, Figure S1: SEM images of CNF. (a) WCNF1, (b) WCNF2; Figure S2: SEM images of BCNF1; Figure S3: The CNF solutions was placed for 1 day and 66 days; Table S1: Different pretreatment methods and preparation of nanocellulose for office waste paper.
