# Supplementary material for: Scalable Preparation of Cellulose Nanofibers from Office Waste Paper by an Environment-Friendly Method
Source: Polymers (Basel). 2021 Sep 15;13(18):3119. doi: 10.3390/polym13183119 (PMC8473219; doi:10.3390/polym13183119)
Supplement: Supplementary file 1 [file polymers-13-03119-s001.zip › polymers-1356569-supplementary.pdf]

Scalable preparation of cellulose nanofibers from office waste paper by an environment-friendly method

Deyuan Huang[a], Haoqun Hong[a], Weilong Huang[a], Haiyan Zhang[a], Xiaobin Hong[b]*

[a] School of Materials and Energy, Guangdong University of Technology, Guangzhou 510006, China.

[b] School of Mechanical and Automotive Engineering, South China University of Technology, Guangzhou 510640, China.

Supporting Information

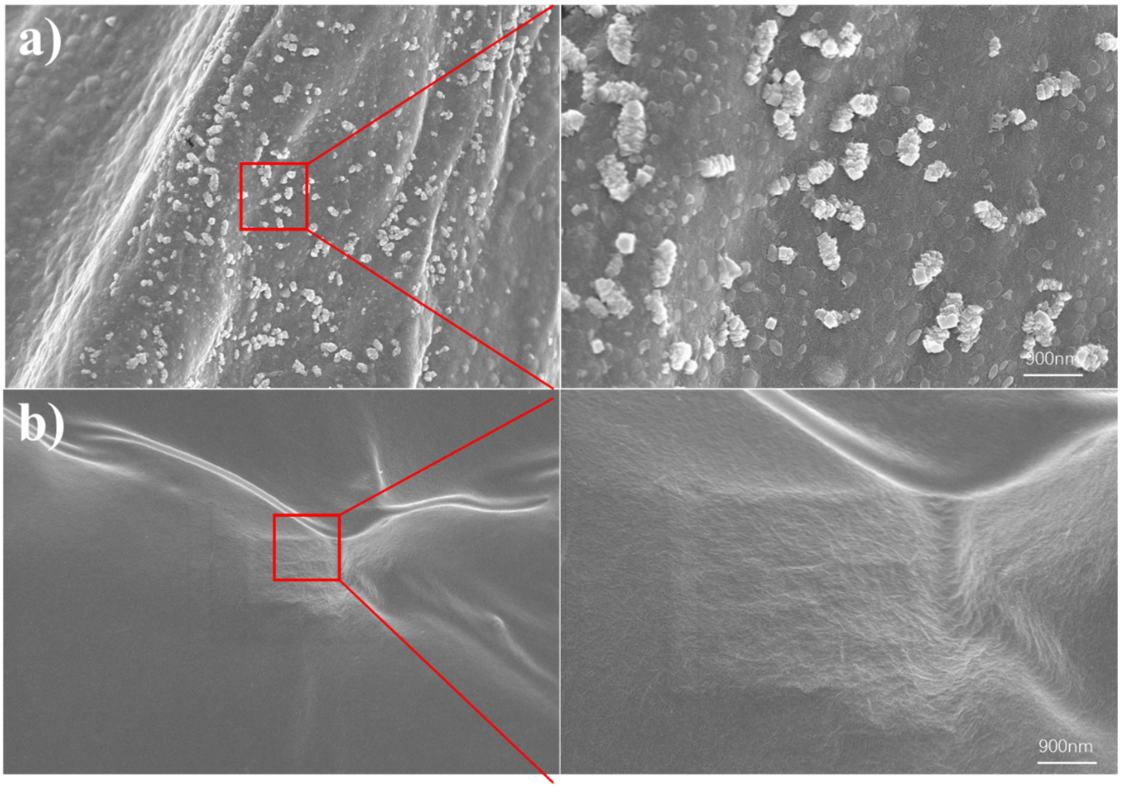

Figure S1. SEM images of CNF. (a)WCNF1, (b)WCNF2

It is obvious that irregular small particles are distributed on the surface of WCNF1, in contrast, the surface of WCNF2 is very smooth in Figure S1.

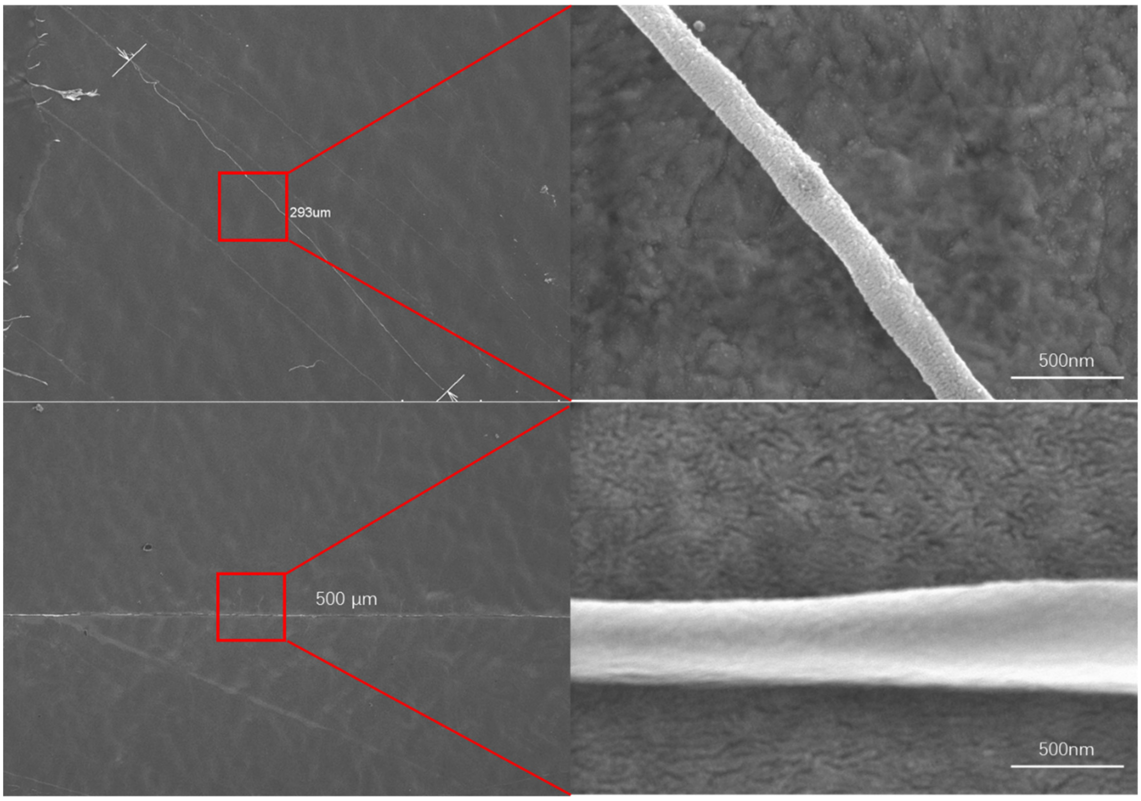

Figure S2. SEM images of BCNF1.

A rough calculation shows that the aspect ratio of BCNF1 is in the range of 1000-1465, indicating that it is possible to prepare CNF with a high aspect ratio by the combination of acid treatment and bleaching treatment.

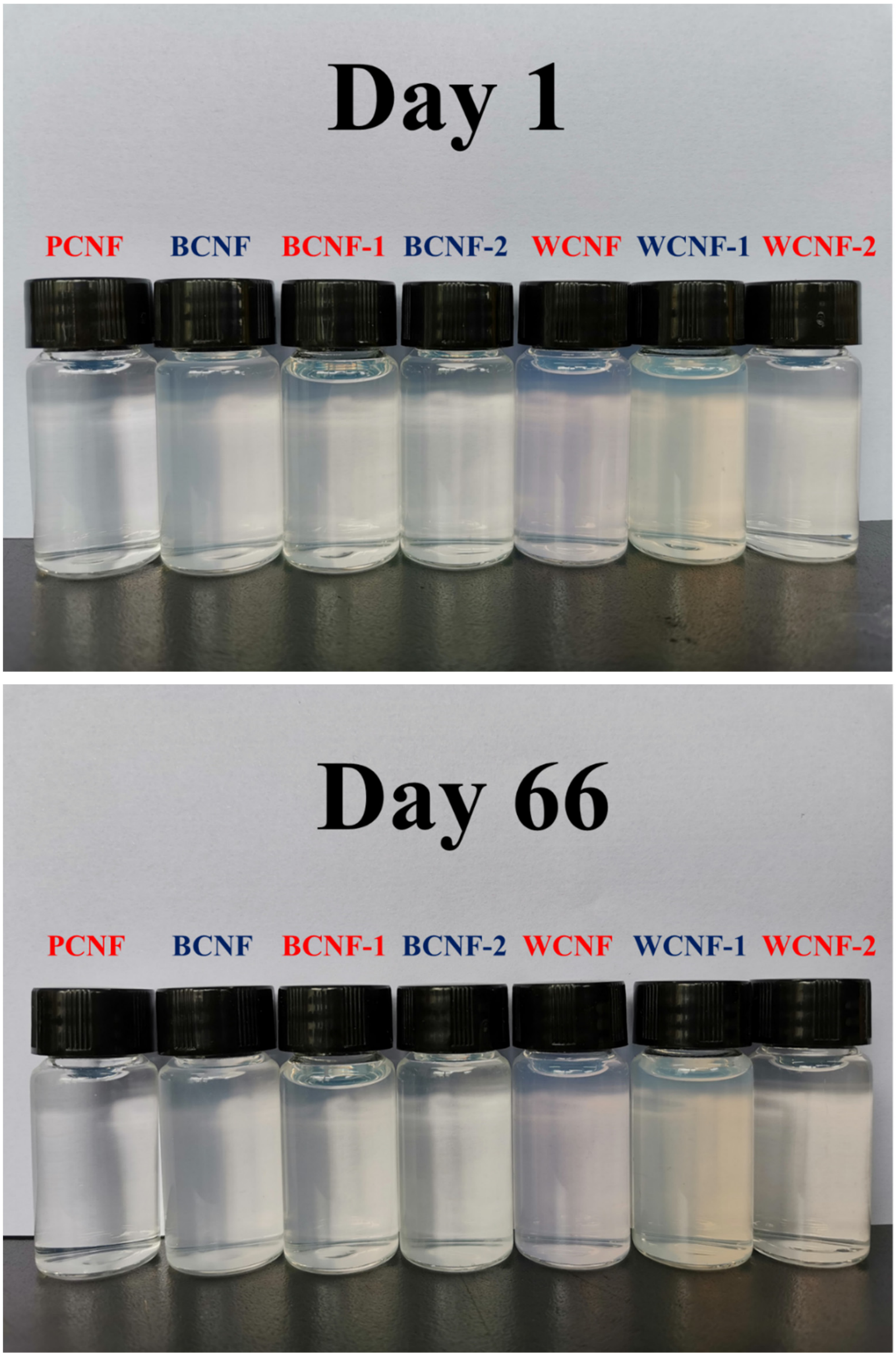

Figure S3. The CNF solutions was placed for 1 day and 66 days.

The CNF solution produced no precipitate after 66 days and showed no significant general change, indicating that the TEMPO-oxidised CNF remained well stabilised at room temperature.

Table S1. Different pretreatment methods and preparation of nanocellulose for office waste paper.

Source	Pretreatment	Type	Fabrication method	Ref.
newspaper	alkali pretreatment (NaOH)<br>bleaching treatment (NaClO)	CNC	60 wt% H_2SO_4 at 45℃, 90 min	[11]
paper residue	alkali pretreatment (NaOH)<br>acid pretreatment (H_2SO_4)<br>bleaching treatments (H_2O_2+ NaOH)	CNC	40 wt% H_2SO_4 at 50℃, 60 min	[12]
waste paper	pulping and flotation pretreatment<br>alkali pretreatment (NaOH)<br>bleaching treatment (H_2O_2+ NaOH)	CNF	ultrafine ground	[13]
newspapers	——	CNF	ultrafine ground	[14]
[a]OWP	bleaching treatment (H_2O_2+ NaOH)<br>deinking pretreatment	CNC	64 wt% H_2SO_4 at 45℃, 60 min	[38]

OWP	deinking pretreatment	CNC	59 wt% H_2SO_4 at 45℃, 60 min	[39]
[b]WTP	——	CNC	47 wt% H_2SO_4 at 60℃, 120 min	[15]
OWP	alkali pretreatment (NaOH)	CNC	64 wt% H_2SO_4 at 45℃, 30 min	[16]
[c]WPP	deinking process (chloroform + dimethyl sulfoxide)<br>alkali pretreatment (NaOH)	CNC	64 wt% H_2SO_4 at 45℃, 60 min	[17]
OWP	——	CNF	mechanical fibrillation	[18]

a: OWP is office waste paper;

b: WTP is waste tissue paper;

c: WPP is waste printing paper
